# MicroNeurotrophins Improve Survival in Motor Neuron-Astrocyte Co-Cultures but Do Not Improve Disease Phenotypes in a Mutant SOD1 Mouse Model of Amyotrophic Lateral Sclerosis

**DOI:** 10.1371/journal.pone.0164103

**Published:** 2016-10-07

**Authors:** Kelly E. Glajch, Laura Ferraiuolo, Kaly A. Mueller, Matthew J. Stopford, Varsha Prabhkar, Achille Gravanis, Pamela J. Shaw, Ghazaleh Sadri-Vakili

**Affiliations:** 1 NeuroEpigenetics Laboratory, MassGeneral Institute for Neurodegenerative Disease (MIND), Massachusetts General Hospital, Boston, MA, 02129–4404, United States of America; 2 Sheffield Institute of Translational Neuroscience (SITraN), University of Sheffield, 385A Glossop Road, Sheffield S10 2HQ, United Kingdom; 3 Department of Pharmacology, School of Medicine, University of Crete, Institute of Molecular Biology & Biotechnology-FORTH, Heraklion 71003, Greece; "INSERM", FRANCE

## Abstract

Amyotrophic Lateral Sclerosis (ALS) is a neurodegenerative disease caused by loss of motor neurons. ALS patients experience rapid deterioration in muscle function with an average lifespan of 3–5 years after diagnosis. Currently, the most effective therapeutic only extends lifespan by a few months, thus highlighting the need for new and improved therapies. Neurotrophic factors (NTFs) are important for neuronal development, maintenance, and survival. NTF treatment has previously shown efficacy in pre-clinical ALS models. However, clinical trials using NTFs produced no major improvements in ALS patients, due in part to the limited blood brain barrier (BBB) penetration. In this study we assessed the potential neuroprotective effects of a novel class of compounds known as MicroNeurotrophins (MNTs). MNTs are derivatives of Dehydroepiandrosterone (DHEA), an endogenous neurosteroid that can cross the BBB and bind to tyrosine kinase receptors mimicking the pro-survival effects of NTFs. Here we sought to determine whether MNTs were neuroprotective in two different models of ALS. Our results demonstrate that BNN27 (10 μM) attenuated loss of motor neurons co-cultured with astrocytes derived from human ALS patients with SOD1 mutations via the reduction of oxidative stress. Additionally, in the G93A SOD1 mouse, BNN27 (10 mg/kg) treatment attenuated motor behavioral impairment in the paw grip endurance and rotarod tasks at postnatal day 95 in female but not male mice. In contrast, BNN27 (10 mg/kg and 50 mg/kg) treatment did not alter any other behavioral outcome or neuropathological marker in male or female mice. Lastly, BNN27 was not detected in post-mortem brain or spinal cord tissue of treated mice due to the rapid metabolism of BNN27 by mouse hepatocytes relative to human hepatocytes. Together, these findings demonstrate that BNN27 treatment failed to yield significant neuroprotective effects in the G93A SOD1 model likely due to its rapid rate of metabolism in mice.

## Introduction

Amyotrophic lateral sclerosis (ALS) is a fatal neurodegenerative disorder characterized by the loss of motor neurons in the cortex, brainstem, and spinal cord. The average age of onset is 50–75 years [1] with patients initially reporting symptoms that include muscle twitching, upper and lower limb weakness, or difficulty swallowing or breathing [1, 2]. ALS patients experience rapid deterioration in muscle function resulting in an average lifespan of 3–5 years following symptom onset [2, 3]. Currently the only disease-modifying therapeutic for ALS, Riluzole, produces modest symptomatic effects prolonging survival by just 3–6 months [4–6], highlighting the critical need for the development of novel therapies that slow or prevent disease progression.

Neurotrophic factors (NTFs) are a class of proteins important for cell differentiation, neuronal growth, and neuronal survival [7]. NTFs include nerve growth factor (NGF), brain-derived neurotrophic factor (BDNF) and neurotrophin 3 (NT3) and 4 (NT4), each of which bind to specific subtypes of pro-survival tyrosine receptor kinases (Trks) as well as p75 neurotrophin receptors [7]. In addition, other growth factors, such as ciliary neurotrophic factor (CNTF) and glial-cell derived neurotrophic factor (GDNF), also produce similar actions in the central nervous system (CNS) [7]. Reduced levels of NTFs have been reported in several neurodegenerative disorders [8], including ALS [9–11], suggesting that loss of trophic support could be important in disease pathophysiology. Additionally, previous studies in ALS mouse models demonstrated preclinical efficacy using BDNF, CNTF, and GDNF [12–22]. As a result, the therapeutic effects of several NTFs were tested in clinical trials for ALS as well as other neurodegenerative disorders [23–26]. Despite the promising preclinical results in ALS models, NTFs have yet to meet their potential as therapies for the treatment of ALS as they did not demonstrate efficacy in clinical trials [27–30]. One major issue with NTF clinical trials was delivery to the CNS. Subsequent studies demonstrated that most NTFs do not readily cross the blood-brain barrier (BBB) [31–34], highlighting a major obstacle underlying the lack of efficacy in NTF clinical trials [35].

Dehydroepiandrosterone (DHEA) is an endogenous neurosteroid produced in both neurons and glia [36, 37] that can also cross the BBB [38]. DHEA, which normally declines with age [39], has been shown to exert neuroprotective effects in several experimental models [40–44]. DHEA is known to alter the activity of γ aminobutiric acid type A (GABA_A_), N-methyl-D-aspartate (NMDA), sigma1, estrogen and androgen receptors at micromolar concentrations [39]. More recent *in vitro* findings demonstrated that DHEA also activates NGF receptors, tyrosine receptor kinase A (TrkA) and p75 neurotrophin receptors, at nanomolar concentrations inducing phosphorylation of TrkA and activation of downstream signaling proteins [45]. Moreover, DHEA treatment attenuated apoptosis in dorsal root ganglia sensory neurons derived from NGF null mice [45] suggesting a neuroprotective role. Based on the promising effects of DHEA, 17-spiro derivatives of DHEA, known as microneurotrophins (MNTs), were synthesized [46]. Similar to DHEA, MNTs exhibit anti-apoptotic effects *in vitro* [46]. While nanomolar concentrations of MNTs bind to and activate TrkA and p75 receptors ([47], Pediaditakis et al., unpublished data), these lower concentrations do not activate steroid hormone receptors [46], thus avoiding the potential estrogenic or androgenic effects of DHEA [39]. Given the neuroprotective effects of DHEA and the structural and functional similarities between DHEA and MNTs, we sought to examine whether the MNTs were neuroprotective in two different models of ALS. First, we measured the effects of MNTs on mouse motor neurons co-cultured with astrocytes derived from human ALS patients with SOD1 mutations. Next, we assessed alterations in body weight, neurological score, motor deficits, and survival in the SOD1 G93A mouse model following treatment with the MNT BNN27. Additionally, neuropathological markers, including lower motor neuron counts in the lumbar spinal cord and quantification of neuromuscular junction (NMJ) integrity in the tibialis anterior muscle, were also assessed following BNN27 treatment.

## Materials and Methods

### MicroNeurotrophin compounds

BNN20, 23 and 27 were characterized previously [46]. Briefly, these were spiro-epoxy derivatives of dehydro-epiandrosterone (DHEA) obtained via modifications at C-17; BNN20: (17β-spiro[5-androstene-17, 20-oxiran]-3β-ol, BNN23: (20S)-3β,21-dihydroxy-17β,20-epoxy-5-pregnene, and BNN27: (20R)-3β,21-dihydroxy- 17R,20-epoxy-5-pregnene. BNN20 is a simple 17,20-spiro epoxy DHEA analogue. BNN23 and BNN27 possess a C21-hydroxyl group, which can participate in hydrogen bond formation with putative molecular targets and thus enhance specific binding capacity to putative receptors. Additionally, BNN23 and BNN27 differ in the stereochemistry of their epoxide ring (BNN23: (20S)-3,21-dihydroxy-17,20-epoxy-5-pregnene, BNN27: (20*R*)-3,21-dihydroxy-17r,20-epoxy-5-pregnene). Additional information in regards to the synthesis of MNTs can be found in [46].

### ES Motor Neuron Differentiation

Mouse embryonic stem cells expressing GFP under the motor neuron (MN)-specific promoter HB9 (HBG3 cells; gift from Tom Jessell) were cultured on primary mouse embryonic fibroblasts (Millipore). For differentiation into MNs, cells were treated with trypsin and resuspended in DFK10 culture medium consisting of knockout DMEM/F12, 10% knockout serum replacement, 1% N2, 0.5% L-glutamine, 0.5% glucose (30% in water), and 0.0016% 2-mercaptoethanol. The cells were plated on non-adherent Petri dishes to allow formation of embryoid bodies. After 1 d of recovery, 2μM retinoic acid (Sigma) and 1μM Smoothened Agonist (SAG) (Millipore) were added to the medium every day for 5 days. Embryoid bodies were then dissociated with papain and sorted using the FACSAria™ III (BD Biosciences).

### Human fibroblasts and co-cultures

Human skin fibroblast samples from 3 unaffected controls and 2 patients with SOD1 mutations (Table 1) were obtained from Prof. Pamela Shaw (University of Sheffield, Sheffield, United Kingdom). Informed written consent was obtained from all subjects before sample collection. These experiments were approved by the Yorkshire & the Humber Research Ethics Committee (study number STH16573). Fibroblasts were reprogrammed to induced neural progenitor cells (iNPCs) and then differentiated into iAstrocytes as previously described [48].

**Table 1:** 

Sample number	Age of onset	Age at Biopsy	Sex	Mutation
91	60	60	M	SOD1 I113T
210	37	42	M	SOD1 D76Y
Control 155	-	40	M	-
Control 3050	-	65	M	-
Control 170	-	63	M	-

iAstrocytes were cultured in DMEM Glutamax (Gibco) with 10% FBS (Sigma) and 0.02% N2 (Invitrogen). On day 0, 10,000 iAstrocytes were seeded in a 96-well plate, on day 1 they were treated with BNN20, 27 and 23 and Riluzole at increasing concentrations of 1, 10 and 30 μM. On day 2, astrocyte medium was removed, cells were washed once with PBS and 10,000 Hb9-GFP^+^ motor neurons were seeded onto the astrocyte monolayer in motor neuron medium (DMEM/F12, 2% knockout serum replacement (Invitrogen), 2% N2, and 2%B27 plus GDNF (Invitrogen; 10 ng/mL), BDNF (Invitrogen; 10 ng/mL), CNTF (Invitrogen; 10 ng/mL)) supplemented with the corresponding concentration of drug. Starting from day 3, each well was scanned using the high content live-imaging system (InCell Bioanalyser) to monitor neuronal survival and axonal growth daily. 9 fields of view at 10x were acquired per well, so that the area scanned covered the entire well. Each condition was repeated in duplicate and each experiment was repeated four times. Data analysis was performed with the InCell Developer software programmed to count only cell bodies with at least one projection (axon), thus guaranteeing that only actual cells and not debris were counted in the experiment.

For the CellRox Assay, iAstrocytes were assayed one day following BNN27 (10 μM) treatment. iAstrocyte monocultures (with or without BNN27) were exposed to CellROX® Orange (Invitrogen), which detects and quantifies reactive oxygen species (ROS) in live cells. In a reduced state, the reagent is cell-permeant and non-fluorescent, but upon oxidation the reagent exhibits strong fluorescence and remains localized within the cell. 30 minutes after applying CellROX, the plate was washed 3 times to eliminate background signal and images were acquired using the InCell system (9 fields per well to cover the whole well). Fluorescence quantification was performed using ImageJ.

### Animals

All animal care, husbandry and experimentation were performed according to the guidelines set by the Massachusetts General Hospital Subcommittee on Research Animal Care. These experiments were approved by the Massachusetts General Hospital Institutional Animal Care and Use Committee (2014N000018). Mice were assessed regularly for motor impairment and euthanized upon onset of major paralysis (see “Weight and Neurological Scoring” section) to minimize suffering. All mice were given access to food and water ad libitum. B6SJL-Tg(SOD1*G93A)1Gur/J male mice [49] were obtained from Jackson Laboratory and bred with C57Bl6 female mice to obtain wild-type (Wt) and mutant transgenic (Tg) G93A SOD1-expressing mice. To determine mouse genotype, DNA extraction was performed from tail biopsies acquired at postnatal day 28–40 followed by quantitative real-time PCR (qRT-PCR) using primers for the mutant G93A SOD1 gene (GGGAAGCTGTTGTCCCAAG and CAAGGGGAGGTAAAAGAGAGC).

### Pellet Implantation

Slow-release 90 d pellets (Innovative Research of America, Inc; Sarasota, Florida) were implanted subcutaneously in the back of mice at postnatal day 55–60. Pellets contained either placebo or BNN27 at 10 mg/kg or 50 mg/kg. Dosing was determined for an average sized male (Wt: 28.5g, Tg: 25.6g) and female (Wt: 23.0g, Tg: 20.0g) mouse.

### Behavior

For all behavioral assessments, the data were collected and analyzed by an investigator who was blind to the experimental treatment condition.

#### Gait analysis

Manual gait analysis was performed using a limb painting procedure similar to previous studies [50, 51]. Mice were first trained to traverse a horizontal corridor leading directly into their home cage by gentle nudges in the appropriate direction if they stopped or attempted to turn around. After pellet implantation, the bottoms of their hindlimbs were painted, by brushing with non-toxic food dye (Fisher Scientific), and the mice were allowed to walk the path to their home cage on a piece of paper. Three trials were performed at each experimental time point (p55, p75, p95, p115, p135). Stride length and width was determined by measuring the distance between the same points, on the ball mount region of the footprint, in two consecutive footprints. Stride length and width was calculated from 2–3 hindpaw strides when the animal was walking continuously at a constant pace. Steps just before the entry to the home cage were not included since mice often slowed down and made smaller steps at this point. Mean data from 4–6 strides across three trials was calculated.

#### Rotarod

Mice were placed on a fixed speed (16 rpm) rotating rod (3.0cm) (Rotamex, Columbus Instruments) [52, 53]. Prior to pellet implantation, mice were trained to remain on the rotarod for 180 seconds. For each experimental time point (p55, p95, p115), the time mice spent on the rotating rod was calculated up to a maximum of 180 seconds. Three trials were performed for each time point and the greatest value for each session was used for analysis.

#### Paw grip endurance test (PaGE)

The PaGE test was performed according to previous studies [53–55]. Briefly, mice were placed on the wire lid of a conventional housing cage that was inverted and held at ~45 cm above an open cage bottom. Prior to pellet implantation, mice were trained to stay on the inverted grid for 90 seconds. For experimental time points (p55, p75, p95, p115, p135), the time spent on the grid (before falling) was noted up to a maximum value of 90 seconds. The largest value from three individual trials was used for analysis.

#### Weight and neurological scoring

Beginning at p55, weight and neurological score (using the ALS TDI criteria) [56, 57] were recorded for each mouse every 5 days until death or euthanasia. ALS TDI criteria are as follows:

Score of 0: Full extension of hind legs away from lateral midline when mouse is suspended by its tail, and mouse can hold this for two seconds, suspended two to three times.
Score of 1: Collapse or partial collapse of leg extension towards lateral midline (weakness) or trembling of hind legs during tail suspension.
Score of 2: Toes curl under at least twice during walking of 12 inches, or any part of foot is dragging along cage bottom/table.
Score of 3: Rigid paralysis or minimal joint movement, foot not being used for generating forward motion.
Score of 4: Mouse cannot right itself within 15 seconds after being placed on either side.

*Mice were euthanized upon obtaining a score of 4.

### Tissue Dissection

Tissue was dissected from Tg G93A SOD1 mice upon reaching a neurological score of 4 [56, 57] or following death (if mice died naturally prior to that point). Mice were sacrificed by administration of slow flow CO_2_ (10–30% of the chamber volume/minute) followed by immediate decapitation. Brain, gastrocnemius, and tibialis anterior tissue were removed and frozen in dry ice. Spinal cord was removed, frozen by gently lowering into the gas byproduct of liquid nitrogen, and dissected into lumbar and non-lumbar regions. All tissue was stored at -80°C prior to use. In addition, tail samples were extracted to perform a second round of confirmatory qRT-PCR for mouse genotyping. Tail samples were stored at -20°C until used.

### Motor neuron quantification

Longitudinal sections (10μm) of the lumbar spinal cord were made from fresh frozen tissue. Hematoxylin and eosin (H&E) staining was performed on the tissue sections. Within the region containing the ventral horn, three sections were counted (each separated by 20–30μm) to cover the areas of highest motor neuron density within the ventral horn. Motor neurons were identified based on their large size (≥15μm) and by the presence of at least one nucleolus. Images were acquired using a Zeiss microscope 20x objective (0.8NA) and processed with Metamorph image analysis software (Molecular Devices).

### Neuromuscular junction quantification

Tissue sectioning, staining, and analysis were performed by Clarapath, Inc (New York, NY). Briefly, longitudinal sections (20μm) of tibialis anterior muscle were made from fresh frozen tissue. One to three sections for each specimen were stained with primary rabbit anti-VACHT (1:10,000; Covance) and secondary Alexa Fluor 488 donkey anti-rabbit (1:500; Life Technologies) antibodies along with a tetramethylrhodamine (TMR) conjugated to α-bungarotoxin (1:750, Life Technologies). Sections were imaged with a 20x objective (0.75NA, 0.45 μm/pixel resolution) and analyzed by in-house software to determine the total number of VACHT and TMR-α-bungarotoxin counts per muscle along with the percent VACHT/TMR-α-bungarotoxin per sample area.

### Mass Spectrometry

Analysis of MNT levels in tissue samples was carried out by Cyprotex (Watertown, MA). Brain and spinal cord samples were homogenized in PBS (2 ml per gram of tissue) and homogenates were crashed with 3 volumes of methanol containing an analytical internal standard (diclofenac). Samples were then centrifuged to remove the precipitated protein and supernatant was analyzed by LC-MS/MS. All brain and spinal cord samples were compared to a calibration curve prepared in mouse blank brain and spinal cord homogenates, respectively. Samples were analyzed by LC-MS/MS using a SCIEX QTrap 5500 mass spectrometer coupled with an Agilent 1290 HPLC Infinity series, a CTC PAL chilled autosampler, both of which were controlled by Analyst software. After separation on a C18 reverse phase HPLC column (Acquity UPLC HSS T3, 1.8, 2.1 x 50 mm) using an acetonitrile-water gradient system, peaks were analyzed by mass spectrometry (MS) using ESI ionization in MRM mode. A quadratic fit calibration curve was used, with a 1/X2 weighting factor applied to the data points. Open symbols were outside of the calibration range and not used in the calculations.

### Statistics

For the *in vitro* iAstroctye-mouse MN co-culture experiments, bar charts are used for graphical representation of the data with the central line representing the mean and the error bar representing the standard error of the mean (SEM). Comparisons were performed using an unpaired t-test or a one-way ANOVA followed by Tukey’s post-tests at a significance level (α) of 0.05.

For the *in vivo* mouse studies, male and female mice were analyzed separately due to their differing body weights and disease onset [58, 59]. Normal distributions of data were not assumed regardless of sample size or variance. Data in the main text are presented as median values. Box plots are used for graphical representation of population data with the central line representing the median, the edges representing the interquartile ranges, and the whiskers representing 10–90th percentiles. Data are also represented as medians ± interquartile ranges or percent values. Sample sizes are included in the figure legends for clarity purpose. Comparisons for unrelated samples were performed using a Mann-Whitney *U* test or a Kruskal-Wallis test followed by Dunn’s multiple comparison post-tests at a significance level (α) of 0.05. For p<0.05 and >0.00001, exact P values (two-tailed) are reported.

## Results

**Fig 1:**
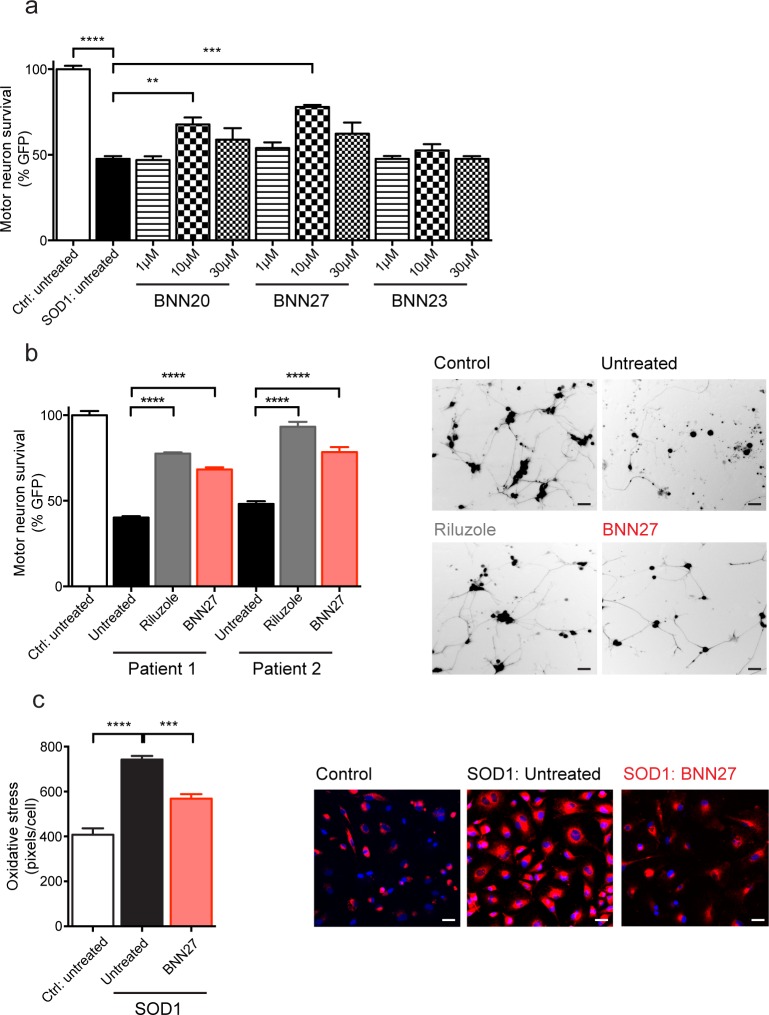
MNTs attenuated survival of mouse motor neurons co-cultured with human astrocytes from SOD1 ALS patients. a) Survival of mouse motor neurons (MNs), expressing GFP under the Hb9 promoter, was significantly decreased in co-cultures with human astrocytes (iAstrocytes) from an ALS patient a SOD1 mutation (Patient 2/SOD1 210; light grey) compared to those from 3 compiled control patients (3 compiled; white) (p<0.0001, unpaired t-test). BNN20, BNN27, and BNN23 were each tested at 1 μM (dark grey), 10 μM (black), and 30 μM (medium grey) in iAstrocyte-mouse MN co-cultures. At 10 μM, BNN20 (p<0.01, Tukey’s test) and BNN27 (p<0.001, Tukey’s test) significantly increased the survival of MNs co-cultured with SOD1 iAstrocytes relative to untreated samples. b) The effects of BNN27 (red) were tested, along with Riluzole (dark grey), on iAstrocytes from 3 control patients (compiled) and 2 individual ALS patients each carrying a distinct SOD1 mutation (Patient 1/SOD1 91 and Patient 2/SOD1 210). Left, BNN27 (10 μM) treatment yielded a significant increase in MN survival in co-cultures with iAstrocytes from Patient 1 (p<0.0001, Tukey’s test) and Patient 2 (p<0.0001, Tukey’s test) relative to untreated co-cultures (light grey). Similarly, Riluzole (10 μM) treatment also significantly increased MN survival in co-cultures with Patient 1 (p<0.0001, Tukey’s test) and Patient 2 (p<0.0001, Tukey’s test) iAstroctyes. Right, representative images of mouse MNs co-cultured with iAstrocytes from control untreated (control), SOD1 210 untreated (untreated), SOD1 210 Riluzole (Riluzole), and SOD1 210 BNN27 (BNN27). Scale bar = 20μM. c) Oxidative stress levels were significantly increased in iAstrocytes from 2 ALS patients containing a SOD1 mutation (Patient 1/SOD1 91 and Patient 2/SOD1 210; light grey) compared to those from 2 control patients (white) (p<0.0001, Tukey’s test). BNN27 treatment (red) produced a significant decrease in oxidative stress levels from SOD1 iAstrocytes relative to untreated cells (p<0.001, Tukey’s test). Scale bar = 10μM. Experiments were performed in triplicate (a, c) and quadruplicate (b). Data are presented as mean ± SEMs. ** p<0.01, ***p<0.001, ****p<0.0001.

To determine which MNT to test in a mouse model of ALS, we assessed the effects of 3 MNTs, BNN20, BNN27, and BNN23 (at 1, 10 and 30 μM), in human astrocytes (iAstrocytes) derived from ALS patients with SOD1 mutations co-cultured with mouse motor neurons (MNs) *in vitro*. In agreement with previous findings [48, 60], MN survival was significantly decreased in co-cultures with iAstrocytes from a SOD1 patient compared to those from control patients (t(4) = 20.23, p<0.0001, unpaired t-test) (Fig 1A). There was an overall effect of MNT treatment on MN survival when co-cultured with SOD1 iAstrocytes (F_(9,20)_ = 0.715, p<0.0001, One-way ANOVA). BNN27 (10 μM) significantly attenuated the motor neuron loss seen in untreated SOD1 iAstrocytes as shown by an increase in MN GFP signal (BNN20- 10 μM: 77.84%, SOD1 untreated: 47.60%; p<0.001, Tukey’s test) (Fig 1A). In addition, BNN20 (10 μM) also attenuated the motor neuron loss seen in untreated SOD1 iAstrocytes (BNN20- 10 μM: 67.76%, SOD1 untreated: 47.60%, p<0.01, Tukey’s test) (Fig 1A).

Since BNN27 (10 μM) yielded the greatest effect on MN survival, we further tested its neuroprotective effects in iAstrocytes derived from two individual ALS patients harboring different SOD1 mutations (SOD1 91 and SOD1 210) (Table 1) along with Riluzole (10 μM), a commonly used ALS therapeutic. Similar to the previous experiments, there was an overall effect of drug treatment on MN survival when co-cultured with SOD1 iAstrocytes (F_(6,21)_ = 2.013, p<0.0001, One-way ANOVA). Specifically, BNN27 (10 μM) treatment yielded a significant increase in MN survival in SOD1 iAstrocytes (SOD1 91- treated: 68.28%, SOD1 91- untreated: 40.22%, p<0.0001, Tukey’s test; SOD1 210- treated: 78.34%, SOD1 210- untreated: 48.13%, p<0.0001, Tukey’s test) similar to the effects of Riluzole (10 μM) treatment (SOD1 91- Riluzole: 77.58%, SOD1 91- untreated: 40.22%, p<0.0001, Tukey’s test; SOD1 210- Riluzole: 93.23%, SOD1 210- untreated: 48.13%, p<0.0001, Tukey’s test) (Fig 1B).

To determine the mechanism of action for BNN27’s neuroprotective effects on MN survival, we assessed oxidative stress levels in iAstrocytes from 2 control patients and 2 SOD1 patients with and without BNN27 treatment. BNN27 treatment in SOD1 iAstrocytes yielded a trend towards a decrease in phospho-p65 (data not shown), the active subunit of NF-κB that mediates inflammatory [61] and oxidative stress [62] pathways. In agreement with these findings, BNN27 treatment yielded an overall effect on oxidative stress in iAstrocytes (F_(2,9)_ = 0.853, p<0.0001, One-way ANOVA) in the CellRox Assay. Specifically, BNN27 treatment significantly decreased reactive oxygen species in SOD1 iAstrocytes (BNN27: 742.5 pixels/cell, SOD1 untreated: 568.3 pixels/cell, p<0.001, Tukey’s test), as indicated by a reduction in fluorescent intensity (Fig 1C).

Given the increased survival of MNs co-cultured with human SOD1 iAstrocytes, following MNT treatment, we next tested the potential neuroprotective effects of BNN27 in the G93A SOD1 mouse model. Both age- and litter-matched wild-type (Wt) and transgenic (Tg; G93A SOD1) male and female mice were implanted subcutaneously with slow release (90 d) pellets (Innovative Research of America, FL) containing BNN27 (10 mg/kg or 50 mg/kg) or placebo. Following pellet implantation alterations in motor behavior outcomes as well as neuropathological markers of disease were assessed.

**Fig 2:**
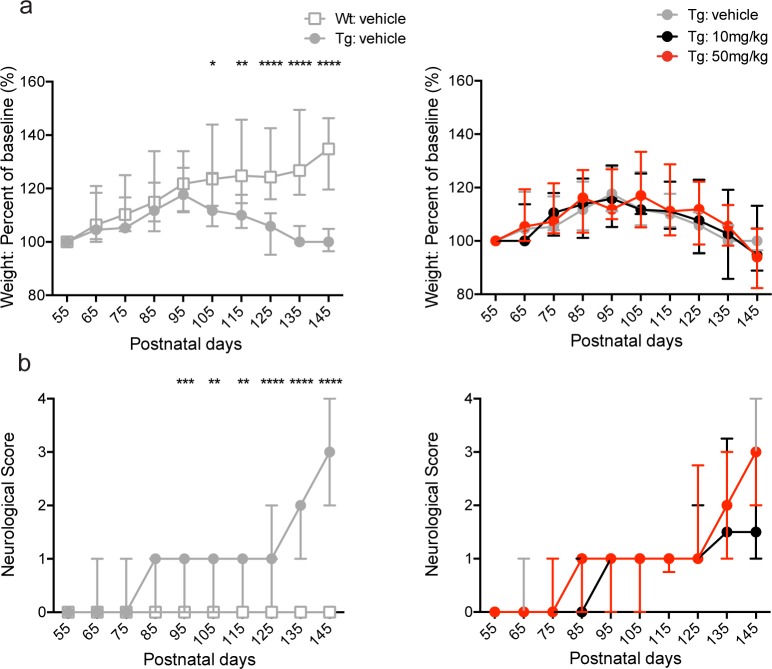
Effect of BNN27 treatment on body weight and neurological score in male and female mice. Tg vehicle-treated mice (males and females combined) exhibited a significant decrease in percent weight relative to baseline levels (a, left) at p105 (p = 0.0188, Mann-Whitney *U* test), p115 (p = 0.0025, Mann-Whitney *U* test), p125 (p<0.0001, Mann-Whitney *U* test), p135 (p<0.0001, Mann-Whitney *U* test), and p145 (p<0.0001, Mann-Whitney *U* test) compared to Wt vehicle-treated mice. Tg vehicle-treated mice also exhibited an increase in neurological score (b, left) at p95 (p = 0.0003, Mann-Whitney *U* test), p105 (p = 0.0036, Mann-Whitney *U* test), p115 (p = 0.0012, Mann-Whitney *U* test), p125 (p<0.0001, Mann-Whitney *U* test), p135 (p<0.0001, Mann-Whitney *U* test), and p145 (p<0.0001, Mann-Whitney *U* test) relative to Wt vehicle-treated mice. There was no significant effect of BNN27 (10 mg/kg or 50 mg/kg) treatment on body weight or neurological score compared to vehicle treatment in Tg mice (p>0.05, Kruskal-Wallis test). Wt-vehicle (n = 20; grey), Tg-vehicle (n = 19; grey), Tg-BNN27 10 mg/kg (n = 20; black), Tg-BNN27 50 mg/kg (n = 18; red). Data are presented as median ± interquartile ranges. * p<0.05, ** p<0.01, ***p<0.001.

First, we determined the effects of BNN27 treatment on body weight and neurological score (see Materials and Methods) [56, 57] in SOD1 mice. As reported previously [52, 53, 63–66], Tg vehicle-treated mice exhibited a decrease in weight gain (data presented as a percent of baseline) at p105 (Tg-vehicle: 111.80%, Wt-vehicle: 123.50%; p = 0.0188, Mann-Whitney *U* test), p115 (Tg-vehicle: 110.00%, Wt-vehicle: 124.80%; p = 0.0025, Mann-Whitney *U* test), p125 (Tg-vehicle: 105.90%, Wt-vehicle: 124.30%; p<0.0001, Mann-Whitney *U* test), p135 (Tg-vehicle: 100.00%, Wt-vehicle: 126.80%; p<0.0001, Mann-Whitney *U* test), and p145 (Tg-vehicle: 100.00%, Wt-vehicle: 134.80%; p<0.0001, Mann-Whitney *U* test) compared to Wt vehicle-treated mice (Fig 2A; left panel). However, BNN27 treatment (10 mg/kg and 50 mg/kg) had no effect on body weight (p>0.05, Kruskal-Wallis test) in Tg mice at any time point tested (Fig 2A; right panel). In addition, Tg vehicle-treated mice exhibited significant motor impairment as indicated by an increase in neurological score [56, 57] at p95 (Tg-vehicle: 1, Wt-vehicle: 0; p = 0.0003, Mann-Whitney *U* test), p105 (Tg-vehicle: 1, Wt-vehicle: 0; p = 0.0036, Mann-Whitney *U* test), p115 (Tg-vehicle: 1, Wt-vehicle: 0; p = 0.0012, Mann-Whitney *U* test), p125 (Tg-vehicle: 1, Wt-vehicle: 0; p<0.0001, Mann-Whitney *U* test), p135 (Tg-vehicle: 2, Wt-vehicle: 0; p<0.0001, Mann-Whitney *U* test), and p145 (Tg-vehicle: 3, Wt-vehicle: 0; p<0.0001, Mann-Whitney *U* test) compared to Wt vehicle-treated mice (Fig 2B; left panel). Similar to the body weight analysis, BNN27 treatment (10 mg/kg and 50 mg/kg doses) had no effect on neurological score (p>0.05, Kruskal-Wallis test) in Tg mice (Fig 2B; right panel).

**Fig 3:**
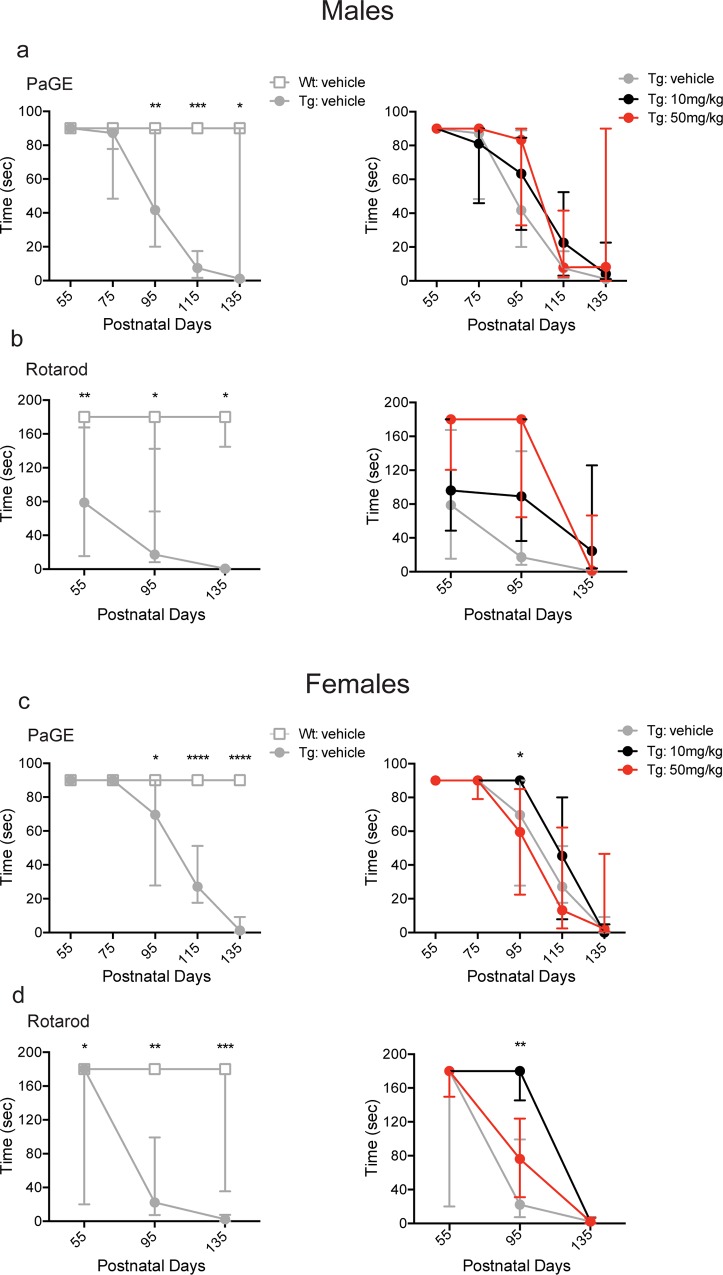
Effect of BNN27 treatment on paw grip endurance (PaGE) and rotarod in male and female mice. Male Tg vehicle-treated mice demonstrated a significant decrease in time spent on the PaGE test (a, left) at p95 (p = 0.0023, Mann-Whitney *U* test), p115 (p = 0.0003, Mann-Whitney *U* test), and p135 (p = 0.0385, Mann-Whitney *U* test) and the rotarod test (b, left) at p55 (p = 0.0089, Mann-Whitney *U* test), p95 (p = 0.0192, Mann-Whitney *U* test), and p135 (p = 0.0152, Mann-Whitney *U* test) relative to Wt vehicle-treated mice. Female Tg vehicle-treated mice demonstrated a decrease in time spent on the PaGE test (c, left) at p95 (p = 0.0128, Mann-Whitney *U* test), p115 (p<0.0001, Mann-Whitney *U* test), and p135 (p<0.0001, Mann-Whitney *U* test) and the rotarod test (d, left) at p55 (p = 0.0379, Mann-Whitney *U* test), p95 (p = 0.0026, Mann-Whitney *U* test), and p135 (p = 0.0002, Mann-Whitney *U* test) relative to Wt vehicle-treated mice. In Tg female mice, BNN27 10 mg/kg treatment significantly increased the time spent on the PaGE (c, right) (p<0.05, Dunn’s multiple comparison test) and rotarod (d, right) (p<0.01, Dunn’s multiple comparison test) tests compared to vehicle treatment at p95. Wt-vehicle (male: n = 10, female: n = 9; grey), Tg-vehicle (male: n = 11, female: n = 11; grey), Tg-BNN27 10 mg/kg (male: n = 9, female: n = 9; black), Tg-BNN27 50 mg/kg (male: n = 9, female: n = 9; red). Data are presented as median ± interquartile ranges. * p<0.05, ** p<0.01, ***p<0.001, ****p<0.0001.

Alterations in muscle strength were assessed following BNN27 or vehicle treatment using the paw grip endurance (PaGE) task. In agreement with previous reports [53, 63, 67], vehicle-treated Tg male mice exhibited deficits in the PaGE task as indicated by decreased time spent on the grid relative to Wt vehicle-treated male mice at p95 (Tg-vehicle: 41.8 sec, Wt-vehicle: 90.0 sec; p = 0.0023, Mann-Whitney *U* test), p115 (Tg-vehicle: 7.4 sec, Wt-vehicle: 90.0 sec; p = 0.0003, Mann-Whitney *U* test), and p135 (Tg-vehicle: 1.1 sec, Wt-vehicle: 90.0 sec; p = 0.0385, Mann-Whitney *U* test) (Fig 3A; left panel). There was no change in performance in the PaGE task following BNN27 treatment (10 mg/kg or 50 mg/kg) compared to vehicle treatment (p>0.05, Kruskal-Wallis test) at any time point tested (Fig 3A; right panel). Similar to males, Tg vehicle-treated females exhibited deficits in the PaGE task as indicated by decreased time spent on the grid relative to Wt vehicle-treated females at p95 (Tg-vehicle: 69.7 sec, WT-vehicle: 90.0 sec; p = 0.0128, Mann-Whitney *U* test), p115 (Tg-vehicle: 27.2 sec, Wt-vehicle: 90.0 sec; p<0.0001, Mann-Whitney *U* test), and p135 (Tg-vehicle: 1.2 sec, Wt-vehicle: 90.0 sec; p<0.0001, Mann-Whitney *U* test) (Fig 3C; left panel). In contrast to males, BNN27 treatment yielded an overall effect in females at p95 in the PaGE task (p = 0.0063, Kruskal-Wallis test). Specifically, BNN27 10 mg/kg treatment increased the time spent in the PaGE task (Tg-10 mg/kg: 90.0 sec, Tg-vehicle: 69.7 sec; p<0.05, Dunn’s multiple comparison test) at p95 relative to vehicle-treated mice (Fig 3C; right panel). However, BNN27 (50 mg/kg) had no effect on PaGE task (p>0.05, Dunn’s multiple comparison test) relative to vehicle-treated female Tg mice at p95. Additionally, there was no change in performance in the PaGE task in female Tg mice treated with BNN27 10 mg/kg or 50 mg/kg at any other time point tested (p>0.05, Kruskal-Wallis test) (Fig 3C; right panel).

Motor coordination was assessed using the rotarod test. Similar to previous findings [52, 53, 63, 65], Tg vehicle-treated male mice demonstrated a decrease in the time spent on the rotarod at p55 (Tg-vehicle: 78.8 sec, Wt-vehicle: 180.0 sec; p = 0.0089, Mann-Whitney *U* test), p95 (Tg-vehicle: 17.2 sec, Wt-vehicle: 180.0 sec; p = 0.0192, Mann-Whitney *U* test), and p135 (Tg-vehicle: 0.5 sec, Wt-vehicle: 180.0 sec; p = 0.0152, Mann-Whitney *U* test) relative to Wt vehicle-treated male mice (Fig 3B; left panel). BNN27 did not alter rotarod performance in Tg male mice treated with either BNN27 10 mg/kg or 50 mg/kg compared to vehicle treatment at any time point assessed (p>0.05, Kruskal-Wallis test) (Fig 3B; right panel). Female Tg vehicle-treated mice also demonstrated a decrease in the time spent on the rotarod at p55 (Tg-vehicle: 180.0 sec, Wt-vehicle: 180.0 sec; p = 0.0379, Mann-Whitney *U* test), p95 (Tg-vehicle: 22.3 sec, WT-vehicle: 180.0 sec; p = 0.0026, Mann-Whitney *U* test), and p135 (Tg-vehicle: 2.4 sec, WT-vehicle: 180.0 sec; p = 0.0002, Mann-Whitney *U* test) relative to Wt vehicle-treated female mice (Fig 3D; left panel). In contrast to the male findings, BNN27 treatment yielded an overall effect on rotarod performance (p = 0.0054, Kruskal-Wallis test) in females. Specifically, BNN27 10 mg/kg treatment in Tg female mice increased the time spent on rotarod (Tg-10 mg/kg: 180.0 sec, Tg-vehicle: 22.3 sec; p<0.01, Dunn’s multiple comparison test) at p95 relative to vehicle-treated Tg female mice (Fig 3D; right panel). However, BNN27 (50 mg/kg) had no effect on rotarod performance (p>0.05, Dunn’s multiple comparison test) relative to vehicle-treated female Tg mice at p95. Additionally, there was no change in rotarod performance in female Tg mice treated with 10 mg/kg or 50 mg/kg BNN27 at any other time point tested (p>0.05, Kruskal-Wallis test) (Fig 3D; right panel).

**Fig 4:**
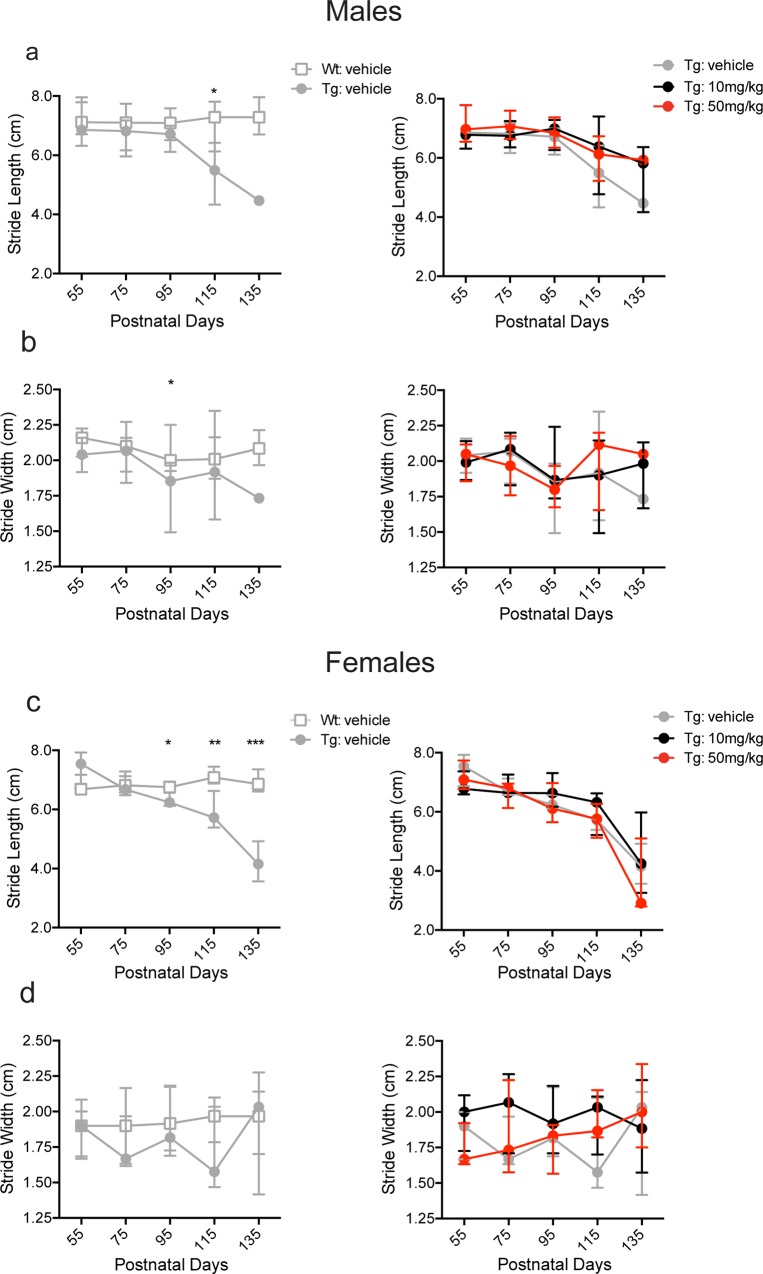
Effect of BNN27 treatment on stride length and width in male and female mice. Male Tg vehicle-treated mice exhibited a decrease in stride length (a, left) at p115 (p = 0.0136, Mann-Whitney *U* test) and stride width (b, left) at p95 (p = 0.0202, Mann-Whitney *U* test) relative to Wt vehicle-treated mice. Female Tg vehicle-treated mice showed a decrease in stride length (c, left) at p95 (p = 0.0155, Mann-Whitney *U* test), p115 (p = 0.0024, Mann-Whitney *U* test), and p135 (p = 0.0010, Mann-Whitney *U* test) but no change in stride width (d, left) (p>0.05, Mann-Whitney *U* test) compared to Wt vehicle-treated mice. There was no significant effect of BNN27 treatment (10 mg/kg or 50 mg/kg) on stride length or width in either male or female Tg mice compared to vehicle treatment (p>0.05, Kruskal-Wallis test) (a-d, right). Wt-vehicle (male: n = 10, female: n = 9; grey), Tg-vehicle (male: n = 8, female: n = 11; grey), Tg-BNN27 10 mg/kg (male: n = 9, female: n = 9; black), Tg-BNN27 50 mg/kg (male: n = 9, female: n = 9; red). Data are presented as median ± interquartile ranges. * p<0.05, ** p<0.01, ***p<0.001.

In addition to the PaGE and rotarod tests, we also performed gait assessment to determine the effects of BNN27 treatment on stride length and width. In agreement with previous studies [49, 63, 64, 66], male Tg vehicle-treated mice demonstrated shortened stride length relative to Wt vehicle-treated males only at p115 (Tg-vehicle: 5.5 cm, Wt-vehicle: 7.3 cm; p = 0.0136, Mann-Whitney *U* test) but not at any other time point tested (p>0.05, Mann-Whitney *U* test) (Fig 4A; left panel). Moreover, stride width was decreased in Tg vehicle-treated males compared to Wt vehicle-treated males at p95 (Tg-vehicle: 1.8 cm, Wt-vehicle: 2.0 cm; p = 0.0202, Mann-Whitney *U* test) but not at any other time point tested (p>0.05, Mann-Whitney *U* test) (Fig 4B; left panel). There was no effect of BNN27 treatment (10 mg/kg and 50 mg/kg doses) on either stride length or width in Tg males (p>0.05, Kruskal-Wallis test) (Fig 4A and 4B; right panel). Female Tg vehicle-treated mice exhibited a decrease in stride length at p95 (Tg-vehicle: 6.2 cm, Wt-vehicle: 6.7 cm; p = 0.0155, Mann-Whitney *U* test), p115 (Tg-vehicle: 5.7 cm, Wt-vehicle: 7.1 cm; p = 0.0024, Mann-Whitney *U* test), and p135 (Tg-vehicle: 4.1 cm, Wt-vehicle: 6.9 cm; p = 0.0010, Mann-Whitney *U* test) compared to Wt vehicle-treated mice (Fig 4C; left panel). However, stride width was not significantly altered in Tg vehicle-treated females relative to Wt vehicle-treated females (p>0.05, Mann-Whitney *U* test) (Fig 4D; left panel). Similar to the males, there was no change in stride length or width in Tg female mice treated with BNN27 10 mg/kg or 50 mg/kg (p>0.05, Kruskal-Wallis test) compared to the vehicle treated group (Fig 4C and 4D; right panel).

**Fig 5:**
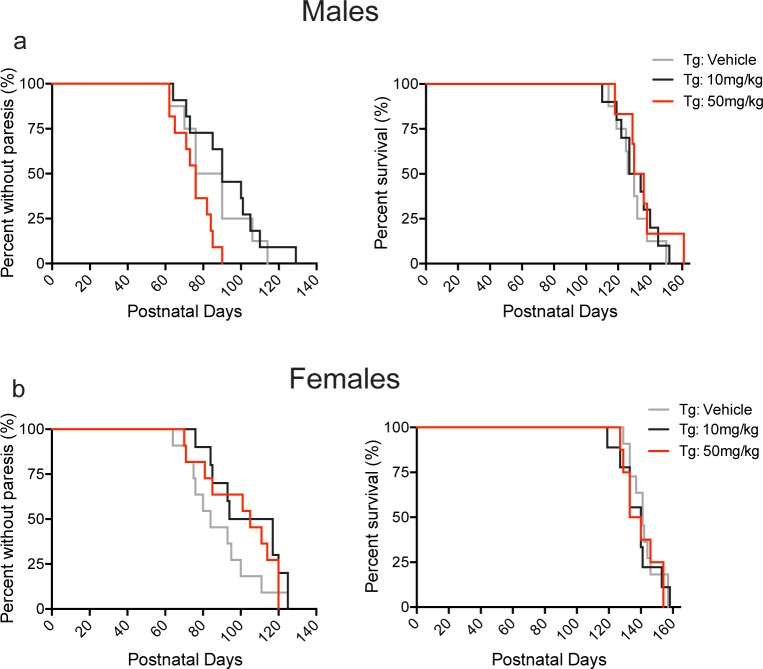
Effect of BNN27 treatment on paresis onset and survival in male and female mice. There was no significant effect of BNN27 (10 mg/kg or 50 mg/kg) treatment on paresis onset or survival compared to vehicle treatment in either male (p>0.05, Kruskal-Wallis test) or female (p>0.05, Kruskal-Wallis test) Tg mice. However, BNN27 10mg/kg treatment in Tg female mice produced a trend (p = 0.0523, Mann-Whitney *U* test) towards an increase in the age of paresis onset relative to Tg vehicle-treated female (b, left). Tg-vehicle (male: n = 8, female: n = 11; grey), Tg-BNN27 10 mg/kg (male: n = 10, female: n = 10; black), Tg-BNN27 50 mg/kg (male: n = 9, female: n = 9; red). Data are presented as percent values.

The effect of BNN27 treatment on the onset of motor symptoms and survival was also assessed. BNN27 10 mg/kg and 50 mg/kg treatment did not significantly alter the age of paresis onset (onset of motor symptoms) (p>0.05, Kruskal-Wallis test) or survival (p>0.05, Kruskal-Wallis test) in male and female Tg mice (Fig 5A and 5B). However, females treated with BNN27 10 mg/kg exhibited a trend towards an increase in the age of paresis onset (Tg-10 mg/kg: 97 days, Tg-vehicle: 84 days; p = 0.0523, Mann-Whitney *U* test) relative to vehicle treatment in Tg female mice (Fig 5B, left panel).

**Fig 6:**
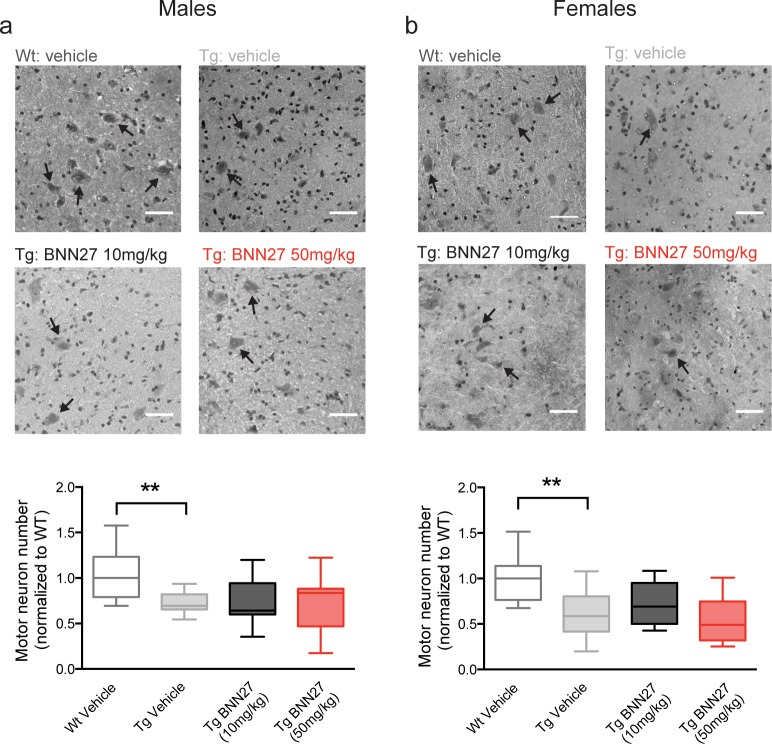
Effect of BNN27 treatment on motor neuron survival in male and female mice. There was no change in motor neuron counts in a) male and b) female mice following BNN27 treatment. Top, representative H&E stained longitudinal sections (10μM) of lumbar spinal cord from Wt vehicle-treated mice and Tg mice treated with vehicle, BNN27 10 mg/kg, or BNN27 50 mg/kg. Arrows indicate motor neurons; scale bar = 150μM. Bottom, quantification of motor neuron counts from Tg mice treated with vehicle (male: n = 7, female: n = 11; grey), BNN27 10 mg/kg (male: n = 8, female: n = 10; black), and BNN27 50 mg/kg (male: n = 9, female: n = 12; red) normalized to Wt vehicle-treated mice (male: n = 12, female: n = 9; white). Tg vehicle-treated mice showed a decrease in the relative motor neuron number compared to Wt vehicle-treated mice in both males (a; p = 0.0095, Mann-Whitney *U* test) and females (b; p = 0.0042, Mann-Whitney *U* test). BNN27 treatment (10 mg/kg or 50 mg/kg) had no effect on motor neuron counts in male (a; p>0.05, Kruskal-Wallis test) or female (b; p>0.05, Kruskal-Wallis test) mice. Data are presented as median and 10-90th percentiles. ** p<0.01.

**Fig 7:**
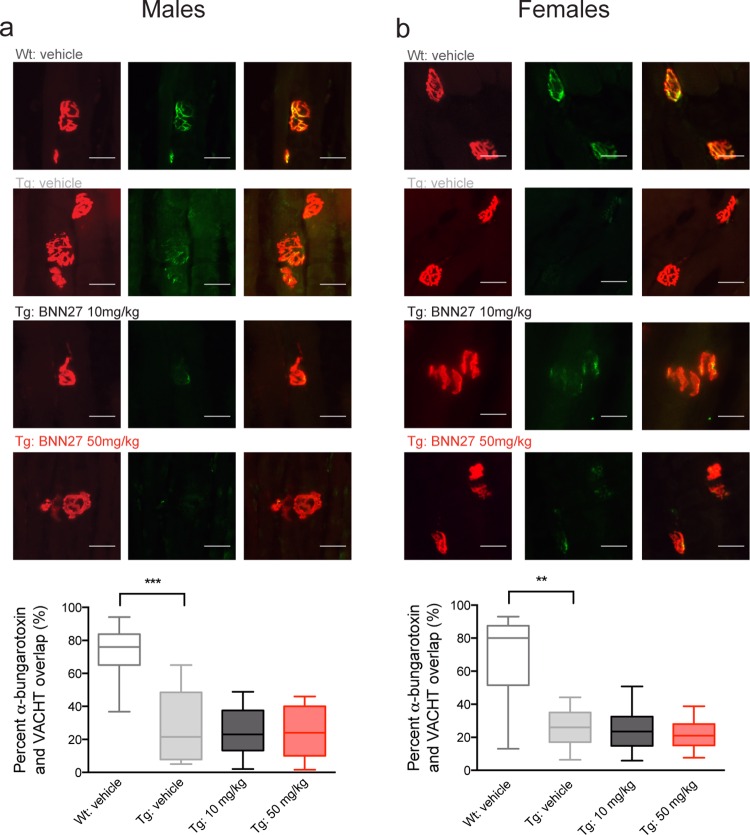
Effect of BNN27 treatment on neuromuscular junction integrity in male and female mice. There was no change in neuromuscular junction (NMJ) integrity in a) male and b) female mice following BNN27 treatment. Top, representative tibialis anterior sections (20μM) from Wt vehicle-treated mice and Tg mice treated with vehicle, BNN27 10 mg/kg or 50 mg/kg. Sections were stained for α-Bungarotoxin (left: TMR-α-Bungarotoxin 1:750; red) and the vesicular acetylcholine transporter (middle: VAChT 1:10,000; green). Overlay images (right) show overlap of α-Bungarotoxin and VAChT. Scale bar = 20μM. Bottom, quantification of the percent overlay between α-Bungarotoxin and VAChT from Tg mice treated with vehicle (male: n = 8, female: n = 11; grey), BNN27 10 mg/kg (male: n = 10, female: n = 10; black) or BNN27 50 mg/kg (male: n = 11, female: n = 13; red) normalized to Wt vehicle-treated mice (male: n = 10, female: n = 9; white). Tg vehicle-treated mice showed a decrease in the relative overlap compared to Wt vehicle-treated males (p = 0.0005, Mann-Whitney *U* test) and females (p = 0.0018, Mann-Whitney *U* test). Data are presented as median and 10-90th percentiles. ** p<0.01, *** p<0.001.

In addition to behavioral analyses, we also assessed the effects of BNN27 treatment on lower motor neuron survival and NMJ integrity. As expected [49, 64, 65], there was a significant decrease in relative motor neuron number in the lumbar spinal cord of Tg vehicle-treated mice compared to Wt vehicle-treated mice in both males (Tg-vehicle: 0.69, Wt-vehicle: 1.00; p = 0.0095, Mann-Whitney *U* test) and females (Tg-vehicle: 0.59, Wt-vehicle: 1.00; p = 0.0042, Mann-Whitney *U* test) (Fig 6). BNN27 treatment did not alter motor neuron survival in male or female Tg mice at 10 mg/kg (p>0.05, Mann-Whitney *U* test) or 50 mg/kg (p>0.05, Mann-Whitney *U* test) compared to vehicle treated mice (Fig 6). Additionally, in agreement with previous findings [68–70], Tg vehicle-treated mice exhibited significant NMJ denervation as indicated by a decrease in the overlap of presynaptic (vesicular acetylcholine transporter; VAChT) and postsynaptic (α-bungarotoxin) markers in the tibialis anterior muscle in both males (Tg-vehicle: 21.50, Wt-vehicle: 76.00; p = 0.0005, Mann-Whitney *U* test) and females (Tg-vehicle: 26.0, Wt-vehicle: 80.0; p = 0.0018, Mann-Whitney *U* test) relative to Wt vehicle-treated mice (Fig 7). There was no significant effect of BNN27 treatment (10 mg/kg or 50 mg/kg) on NMJ denervation in both male (p>0.05, Mann-Whitney *U* test) and female (p>0.05, Mann-Whitney *U* test) mice (Fig 7).

Upon termination of the behavioral and neuropathological analyses, we assayed the bioavailability of BNN27 in post-mortem tissue. BNN27 was not detected in post-mortem brains or spinal cords of treated mice (S2 Fig), in agreement with recent data from our consortium demonstrating that BNN27 is rapidly metabolized in mouse compared to human hepatocytes [71]. Together, these findings demonstrated that BNN27 does not significantly alter the disease onset or progression in the G93A SOD1 mouse model of ALS. However, the rapid decay kinetics of BNN27 in the mouse along with the neuroprotective effects of BNN27 in iAstrocyte-mouse MN co-cultures suggest that human-derived cell lines or larger animals may provide a more appropriate model for testing MNT efficacy in ALS.

## Discussion

In the current study, the neuroprotective effects of the MNT BNN27, a novel DHEA derivative, were assessed in mouse motor neurons co-cultured with human astrocytes from ALS patients with a SOD1 mutation and in the G93A SOD1 mouse model of ALS. Our findings demonstrate that *in vitro* BNN27 (10 μM) attenuated loss of motor neurons co-cultured with human SOD1 iAstrocytes via the reduction of oxidative stress. Additionally, in the G93A SOD1 mouse, BNN27 (10 mg/kg) treatment attenuated motor behavioral impairment in the PaGE and rotarod tasks at p95 in female but not male mice. However, BNN27 (10 mg/kg and 50 mg/kg) treatment did not affect body weight, neurological score, gait, the age of onset of paresis or survival in male or female mice. In addition, lumbar spinal cord motor neuron number and tibialis anterior NMJ integrity were not altered following BNN27 treatment (10 mg/kg and 50 mg/kg) in male or female mice. Moreover, our results demonstrate that BNN27 was not detected in the brain or spinal cord of treated mice, in agreement with *in vitro* experiments from our consortium illustrating that BNN27 was quickly metabolized by mouse hepatocytes [71]. Together, these data demonstrate that BNN27 treatment failed to produce significant neuroprotective effects in the G93A SOD1 mice likely due to its rapid rate of metabolism in mice.

In this study, we demonstrated that BNN27 reduced MN cell loss in *in vitro* co-cultures at least in part via a decrease in oxidative stress. Oxidative stress has been shown to play a major role in several neurodegenerative diseases, including ALS. Previous work from our group demonstrated that overexpression of mutant SOD1 in NSC34 cells resulted in a suppression of the cellular antioxidant response [72], thus identifying oxidative stress as one of the toxic mechanisms leading to MN cell death. Our current findings are consistent with previous data demonstrating that therapeutic approaches that enhance the anti-oxidant cellular response in astrocytes are beneficial and protective in the G93A SOD1 mouse [73].

In the current study, we found that BNN27 treatment (10 mg/kg) significantly improved performance in the PaGE and rotarod tasks in female mice only at p95 (Fig 4C and 4D, right panel). These data suggest a sex-specific effect of drug treatment in female G93A SOD1 mice, which has not been previously reported. Prior studies have described a more significant therapeutic improvement in female relative to male G93A SOD1 mice [74, 75]. However, previous reports describing sex-specific effects of therapeutic treatment have been limited to male G93A SOD1 mice only [76–83]. In addition, disparate disease courses have been described in male and female G93A SOD1 mice even when age- and litter-matching occurred. Specifically, female G93A SOD1 mice exhibit prolonged survival compared to male cohorts [58, 59], in agreement with the findings from our present study (S1 Fig). Moreover, when litter-matching is employed, there is decreased noise within a female-only cohort, compared to a male-only cohort [59]. An alternative explanation for these findings is the possible interaction of BNN27 with the estrogen receptor (ER). Although previous *in vitro* data demonstrate that MNTs do not activate hormone receptors at nanomolar concentrations [46], in this study, BNN27 was administered continuously via slow release pellets at 10 and 50 mg/kg, doses, which may lead to the activation of ER or other hormone receptors. Together these sex-specific differences may contribute to the significant behavioral improvement seen in female but not male G93A SOD1 mice in the current study.

Our findings illustrate that the G93A SOD1 mutant mice exhibit a high degree of behavioral variability, even within age-matched and litter-matched sample groups, which agrees with previous findings [58, 59]. Therefore, in accordance with published guidelines [59, 84] we aimed for large samples sizes (~24 gender-balanced mice per experimental group) for this study but ended up fewer mice than initially anticipated (18–20 gender-balanced mice per experimental group) due to several factors. Several mice were omitted from the study due to pellet implantation issues for the BNN27 50 mg/kg group. As expected, the 50 mg/kg pellets were larger in size than the placebo or 10 mg/kg pellets. Therefore, the pellets partially or completely fell out in some mice that were subsequently excluded from the study. Moreover, a few mice died during the study from issues unrelated to the drug treatment or the normal disease course in G93A SOD1 mice and were thus excluded from the study in accordance with the recommended guidelines for this mouse line [57, 59]. Therefore, the reduced sample sizes could in part explain the lack of effect of BNN27 treatment on Tg SOD1 mice and future studies with these compounds might benefit from a larger sample size.

The major confound in this study was the bioavailability and metabolism of BNN27. Mass-spectrometry results demonstrated that BNN27 was not detected in post-mortem brain or spinal cord tissue from BNN27 treated mice (S2 Fig). In contrast, BNN27 was detected in post-mortem mouse brain tissue at 30 min following an acute i.p. injection of BNN27 (50 mg/kg) [71]. The discrepancy between these data could be due to issues with the pellet formulation or timing of drug administration with the implanted pellets relative to when tissue was collected. Additionally, at 30 min following an i.p. injection of BNN27 (50 mg/kg), two hydroxylated metabolites of BNN27 were also present in post-mortem brain tissue [71]. However, the function of these metabolites is not yet known. It is possible that these BNN27 metabolites could bind to TrkA receptors and activate anti-apoptotic downstream signaling cascades similar to NGF or DHEA. However, the modest behavioral improvements seen in this study (only in females at p95) suggest that these metabolites are not activating the same proposed downstream signaling cascades, of DHEA-like compounds, to exert neuroprotective effects. Regardless, further *in vitro* studies are warranted to determine the function and downstream effects of these hydroxylated metabolites of BNN27.

To assess the metabolic profile of BNN27, our consortium performed *in vitro* studies with human and mouse hepatocytes [71]. The same two hydroxylated BNN27 metabolites, detected after i.p. injection, were found following BNN27 incubation with both mouse and human hepatocytes [71]. However, the half-life of BNN27 was markedly shorter in experiments using mouse hepatocytes relative to human hepatocytes [71]. These data suggest that BNN27 may be largely metabolized before entering the CNS in mice and explain the absence of BNN27 in the brain and spinal cord of treated mice. The longer half-life of BNN27 in human hepatocytes suggests that BNN27 may provide neuroprotective effects in human ALS cell models. However, this area requires further exploration.

Human induced pluripotent stem (iPS) cells and induced neural progenitor cells (iNPCs) from ALS patients have been used to model disease phenotypes and assess drug outcomes [48, 85–89]. Specifically, human ALS astrocyte-mouse motor neuron co-cultures have been previously employed to test potential therapeutic approaches [60, 90] similar to the approach taken in our current study (Fig 1). Therefore, future studies building on our current findings in patient-derived cells (Fig 1) could provide a more appropriate model for testing the potential efficacy of these novel DHEA derivatives. Moreover, future BNN27 metabolism studies in hepatocytes from other large animals (e.g. dogs, pigs) could provide important information regarding the metabolism profile of BNN27. In conclusion, our study was not able to assess the neuroprotective efficacy of BNN27 *in vivo* as the parent MNT was not detected in the CNS. Thus, more extensive and additional studies in other ALS models are warranted in order to justify the potential use of BNN27 as a novel treatment in human ALS patients.

## Supporting Information

S1 Fig Survival in male and female Tg SOD1 mice. Female Tg vehicle-treated mice exhibited prolonged survival (female: 142 d, n = 10; male: 128 d, n = 8; p = 0.0190, Mann-Whitney *U* test) relative to male Tg vehicle-treated mice. * p<0.05.
(PDF)

S2 Fig BNN27 levels in post-mortem brain and spinal cord tissue from treated mice. BNN27 was not present in post-mortem a) brain or b) spinal cord following chronic pellet implantation. A representative LC-MS/MS graph showing that BNN27 levels were undetectable (below the limit of quantification) in post-mortem a) brain and b) spinal cord from pellet (50 mg/kg) implanted mice.
(PDF)
