# Supplementary figures and images for: MicroNeurotrophins Improve Survival in Motor Neuron-Astrocyte Co-Cultures but Do Not Improve Disease Phenotypes in a Mutant SOD1 Mouse Model of Amyotrophic Lateral Sclerosis
Source: PLoS One. 2016 Oct 7;11(10):e0164103. doi: 10.1371/journal.pone.0164103 (PMC5055348; doi:10.1371/journal.pone.0164103)

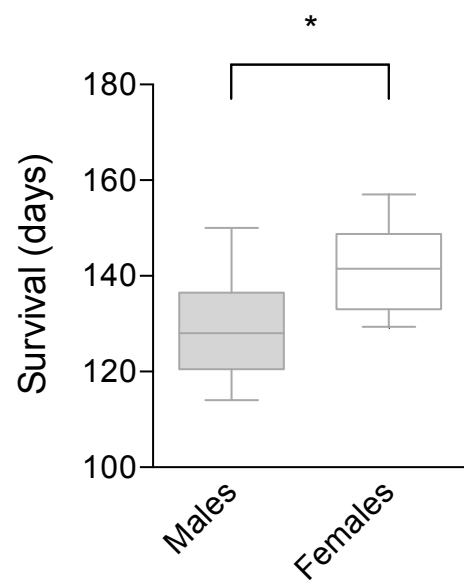

Supplement: S1 Fig — Female Tg vehicle-treated mice exhibited prolonged survival (female: 142 d, n = 10; male: 128 d, n = 8; p = 0.0190, Mann-Whitney U test) relative to male Tg vehicle-treated mice. * p<0.05. (PDF) [file pone.0164103.s001.pdf]
