# Supplementary material for: MicroNeurotrophins Improve Survival in Motor Neuron-Astrocyte Co-Cultures but Do Not Improve Disease Phenotypes in a Mutant SOD1 Mouse Model of Amyotrophic Lateral Sclerosis
Source: PLoS One. 2016 Oct 7;11(10):e0164103. doi: 10.1371/journal.pone.0164103 (PMC5055348; doi:10.1371/journal.pone.0164103)

a

Acute i.p. injection: brain

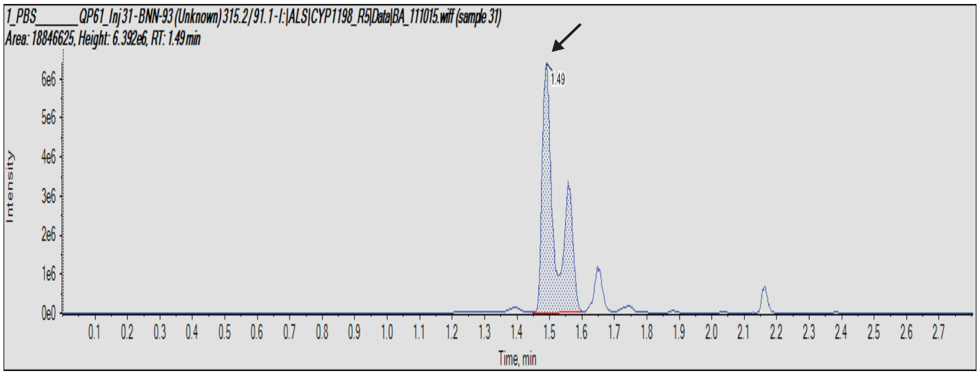

b

Chronic pellet injection: brain

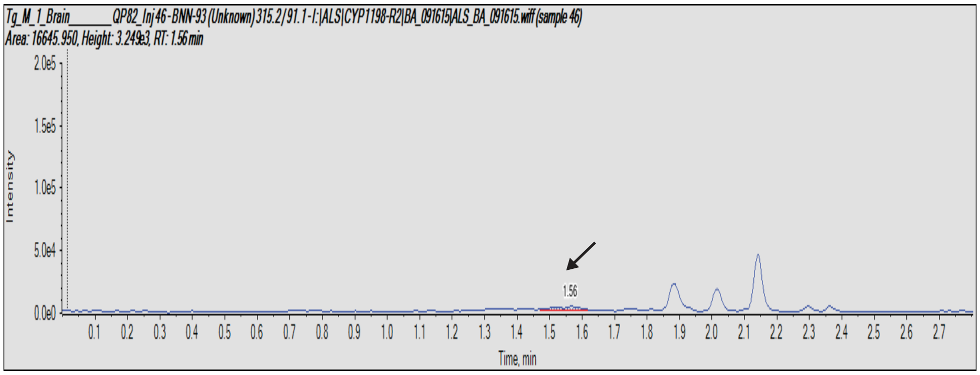

c

Chronic pellet injection: spinal cord

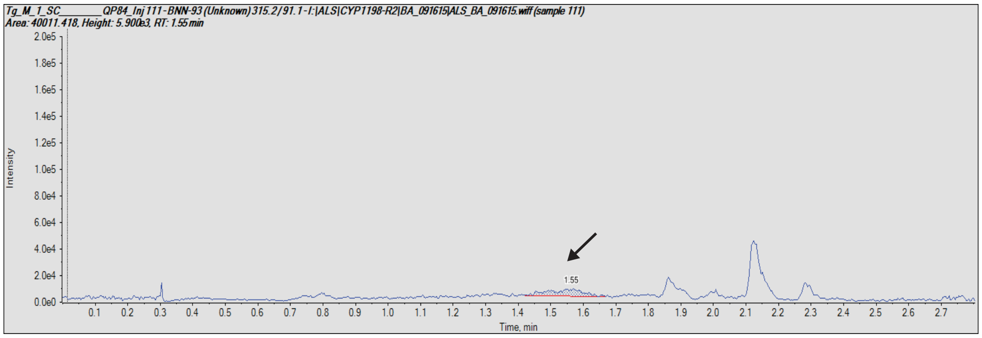

Supplement: S2 Fig — BNN27 was not present in post-mortem a) brain or b) spinal cord following chronic pellet implantation. A representative LC-MS/MS graph showing that BNN27 levels were undetectable (below the limit of quantification) in post-mortem a) brain and b) spinal cord from pellet (50 mg/kg) implanted mice. (PDF) [file pone.0164103.s002.pdf]
